# Prenatal epigenetics diets play protective roles against environmental pollution

**DOI:** 10.1186/s13148-019-0659-4

**Published:** 2019-05-16

**Authors:** Shizhao Li, Min Chen, Yuanyuan Li, Trygve O. Tollefsbol

**Affiliations:** 1 Department of Biology, University of Alabama at Birmingham, Birmingham, AL USA; 2 Department of Pharmacology and Toxicology, University of Alabama at Birmingham, Birmingham, AL USA; 3 Comprehensive Cancer Center, University of Alabama at Birmingham, Birmingham, AL USA; 4 Nutrition Obesity Research Center, University of Alabama at Birmingham, Birmingham, AL USA; 5 Comprehensive Center for Healthy Aging, University of Alabama at Birmingham, Birmingham, AL USA; 6 Comprehensive Diabetes Center, University of Alabama at Birmingham, Birmingham, AL USA

**Keywords:** Epigenetics diet, Environmental pollution, DNA methylation, Histone modification, miRNA

## Abstract

It is thought that germ cells and preimplantation embryos during development are most susceptible to endogenous and exogenous environmental factors because the epigenome in those cells is undergoing dramatic elimination and reconstruction. Exposure to environmental factors such as nutrition, climate, stress, pathogens, toxins, and even social behavior during gametogenesis and early embryogenesis has been shown to influence disease susceptibility in the offspring. Early-life epigenetic modifications, which determine the expression of genetic information stored in the genome, are viewed as one of the general mechanisms linking prenatal exposure and phenotypic changes later in life. From atmospheric pollution, endocrine-disrupting chemicals to heavy metals, research increasingly suggests that environmental pollutions have already produced significant consequences on human health. Moreover, mounting evidence now links such pollution to relevant modification in the epigenome. The epigenetics diet, referring to a class of bioactive dietary compounds such as isothiocyanates in broccoli, genistein in soybean, resveratrol in grape, epigallocatechin-3-gallate in green tea, and ascorbic acid in fruits, has been shown to modify the epigenome leading to beneficial health outcomes. This review will primarily focus on the causes and consequences of prenatal environment pollution exposure on the epigenome, and the potential protective role of the epigenetics diet, which could play a central role in neutralizing epigenomic aberrations against environmental pollutions.

## Background

A report by the World Health Organization (WHO) estimated that 1.8 billion children (around 93% of the world’s children) breathe polluted air every day, leading to 600,000 children who died from acute lower respiratory infections in 2016. Another recent set of data from WHO shows that in 2012, environmental risk factors, such as air, water and soil pollution, chemical exposures, climate change, and ultraviolet radiation caused 12.6 million deaths, which involve more than 100 diseases and injuries. Accumulating evidence strongly suggests that environmental pollution is seriously affecting human health.

Epidemiological studies suggest that early life, especially prenatal, exposure to environmental factors can induce persistent metabolic and physiological changes in the fetus through the altered epigenetic profiles leading to different susceptibility to various chronic diseases such as obesity, cardiovascular, diabetes, and even cancer in later life. Epigenetics refer to mitotically or meiotically heritable changes in gene expression without a change in the DNA sequence [1, 2]. It was first defined by Conrad Waddington in the 1940s as “…the interactions of genes with their environment which bring the phenotype into being” [3], which provides a potential mechanism through which the environmental factors interact with intrinsic factors and have an impact on gene regulation. Certain chemical modifications to DNA, histone protein and RNA, and non-coding RNAs form a complex regulatory network that modulates the chromatin conformation and gene expression. DNA methylation generally refers to a process by which methyl groups are added to the 5-carbon of the cytosine ring resulting in 5-methylcytosine (5mC). DNA methylation is almost exclusively found in CpG sites, which are regions of DNA where a cytosine nucleotide occurs next to a guanine nucleotide in the liner sequence of bases along its length, in mammals [4]. Histone modifications are a diverse array of post-translational modifications that often occur on tail domains of histone proteins, including acetylation, phosphorylation, methylation, ubiquitination, sumoylation, and adenosine diphosphate (ADP)-ribosylation [5]. The epigenome refers to the complete description of all these potentially heritable changes across the genome [6], among which DNA methylation and covalent modifications of histones are the most important epigenetic regulations that have been well studied.

Mammalian embryos experience two major cycles of epigenetic reprograming including the periods of germ cell development and preimplantation, during which the epigenome is vulnerable to endogenous and exogenous environmental factors. The perturbation of prenatal epigenome reprograming has been shown to influence disease susceptibility in the offspring. The Fetal Basis of Adult Disease (FEBAD) hypothesis postulates that prenatal insults such as nutrition or environmental stimulation can disturb developmental programming leading to a higher risk of disease in later life [7]. The Developmental Origins of Health and Disease (DOHaD), another similar concept that is used to describe developmental plasticity, points to the critical role of environmental cues during the transfer from genotype to phenotype [8, 9]. Recently, the focus of DOHaD has extended from poor in utero nutrition to non-nutritional factors that may influence organism’s physiology, thus changing disease susceptibility in adulthood. Among these non-nutritional risk factors, early-life exposure to environmental contaminants attracts considerable attention.

Accumulating studies propose that epigenetics may be one of the most important molecular mechanisms linking environmental stimulation, fetal programming, and adulthood phenotype. Due to their reversible nature, epigenetic modifications are becoming an attractive therapeutic target [2]. An increasing body of evidence shows that maternal diets are associated with persistent metabolic changes in the offspring and can substantially improve the health of children and adults, which is referred to as nutritional programming. In this context, nutritional epigenetics emerge and provide a novel way to prevent developmental perturbation by environmental factors. The epigenetics diet, a term coined by our lab in 2011, refers to a class of bioactive dietary compounds that can regulate the epigenome [10]. Studies indicate that the epigenetics diet plays a critical role in epigenetic regulation such as DNA methylation, histone modification, and microRNA (miRNA) regulation. Some bioactive compounds may counteract or attenuate the damage to the epigenome caused by pollution. As a most striking example, it has been shown that maternal supplementation with methyl donors can reverse DNA hypomethylation induced by bisphenol A, an endocrine-disrupting chemical of public health concern [11].

The purpose of this review is to provide a summary of recent research findings on the influence and causes of early life, especially prenatal exposure, to environmental contaminants on the epigenome, and the potential mechanisms through which parental epigenetic dietary supplementation prevents environment pollution-induced adverse effects. Our review will provide implications into new preventive and therapeutic strategies for coping with increasingly severe environmental pollution.

## Epigenetic stability during gametogenesis and embryonic development

**Fig. 1:**
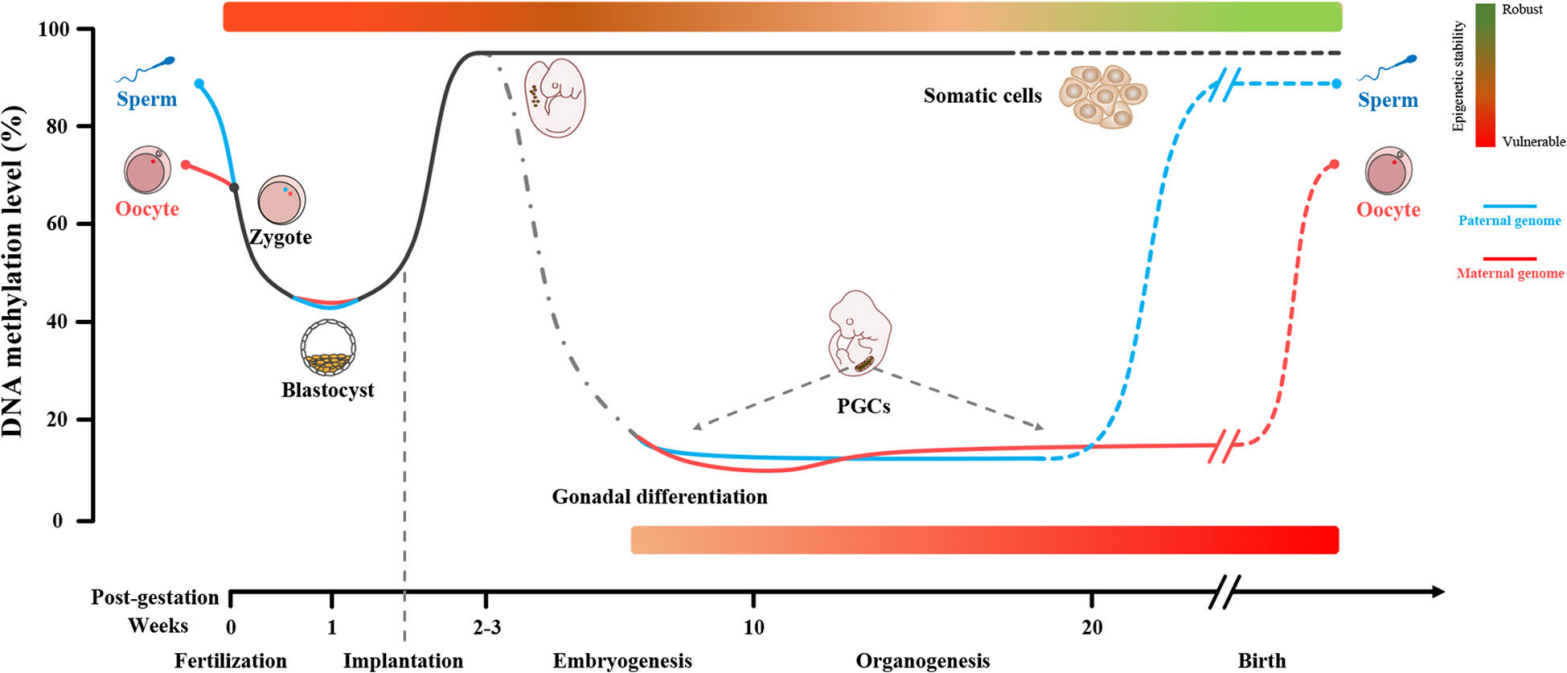
Schematic of DNA methylation dynamic and epigenetic stability during gametogenesis and embryogenesis in humans. DNA methylome reprogramming in germ cells: primordial germ cells (PGCs) in the human become demethylated early in development; from gonadal differentiation to gametogenesis, DNA methylation of spermatocyte and oocyte cells rises gradually until fertilization, at which point sperm reaches a higher methylation level than the oocyte. DNA methylome reprogramming during embryonic development: after fertilization, within the first week, the methylation level of the zygote decreases to the lowest level at the early blastocyst period, during which DNA methylation of the paternal genome reaches a lower level; subsequently, de novo methylation occurs in somatic cell lineages, until they develop into mature somatic cells with stable methylation levels. Epigenetic stability during development: epigenetic stability is proportional to DNA methylation levels. The blue line represents the paternal genome. The red line indicates maternal genome. The gray dashed line denotes mature somatic cells. From red to green, signifies from vulnerable to robust of the epigenome. PGCs, primordial germ cells. Adapted and used with permission from Guo et al. [14], Hemberger and Pedersen [46] and Zhu et al. [20]

The mammalian embryo undergoes two large-scale waves of epigenomic reprogramming (Fig. 1): the first wave takes place from sperm-egg fusion to preimplantation; the second wave happens during genesis of germ cells [12, 13]. Here, we review the DNA methylome, which is one of the most important components in the epigenome, reprogramming in mammals, and its susceptibility to the environment.

### Epigenomic reprogramming during early embryogenesis

After fertilization, a dramatic demethylation takes place in the early embryo. The amount of methylation in sperm (86%, median) and in oocyte (72%, median) rapidly reduce and reach their minimum level (43%, median) in the inner cell mass (ICM) of the early blastocyst-stage embryos (32–64 cells) [14, 15]. In this process, early gamete-specific methylation patterns inherited from the parents as well as acquired epigenetic modifications are erased, while methylated regions in imprinted genes are accurately retained, which is crucial to pluripotency establishment. For instance, the imprinted genes *H19* [16] and *Rasgrf1* [17] in the paternal genome are protected from extensive demethylation after fertilization [18], under the action of DNA methyltransferase 1 (DNMT 1) [19]. Before genome-wide demethylation, remarkable transformation of the paternal genome occurs in the egg cytoplasm, where protamine of sperm chromatin is replaced by acetylated histones, suggesting that demethylation may be linked to chromatin remodeling [18]. A recent study found that after the two-cell stage, demethylation occurring in the paternal genome is much faster and thorough than that in the maternal genome, and this higher maternal genome methylation is maintained until the post-implantation stage, especially in the extra-embryonic villus [20, 21]. This finding indicates that the DNA methylome is asymmetrically distributed in the maternal and paternal genomes. Conversely, compared to the paternal genome, the maternal genome may contribute more DNA methylation memory to the early embryo; thus, adverse environmental factors such as pollutants, virus and malnutrition are more likely to change methylation patterns in the maternal genome during gametogenesis leading to acute dysplasia and disease susceptibility in later life. It is noted that demethylation and de novo methylation keep dynamic equilibrium before global methylation of the early embryo reaching the lowest level at the blastula stage [20]. After implantation, the first wave of de novo methylation occurs [1], and DNA methylation patterns are reestablished by DNMTs in the blastocyst stage. Curiously, however, the timing of remethylating the paternal epigenome is earlier than the maternal one, among which *H19* is a typical example [22]. At about 2–3 weeks of age, the cells in the human embryo are gradually developing into mature somatic cells with relatively stable methylation levels [14] (Fig. 1).

### Epigenomic reprogramming during gametogenesis

Human primordial germ cells (PGCs) are produced in the early stage of embryonic development. PGCs are the embryonic progenitors of oocytes and sperm [15], which can transmit genetic information to offspring and maintain the continuance of the species. Under normal circumstances, DNA methylation markers on genomic DNA of most tissues and organs in the post-implantation embryo will remain basically stable, whereas the DNA methylome in PGCs will experience the second massive elimination and reconstruction, which is much more thorough than the first wave in preimplantation embryos [14]. Approximately 10–11 weeks after gestation, the global DNA methylation of PGCs are drastically decreased from over 90% in the early post-implantation embryo to only 7.8% and 6.0% in male and female PGCs, respectively [14]. Although DNA methylation memory in most regions of PGCs is completely erased, some repetitive sequences still retain higher levels of residual methylation, especially the evolutionarily younger sequences and the alpha satellites [14, 23, 24], suggesting a basis for potential transgenerational epigenetics. After a period of hypomethylation, remethylating of the male germ cells takes place during late embryonic development, whereas de novo methylation in the female germ cells occurs after birth, due to a protracted developmental process. It has been shown that global DNA methylation of human sperm cells is higher than that in oocytes [14]. There are three likely purposes of reprogramming in germ cells: resetting of imprints, which mainly occurs in species with imprinting [18]; removal of acquired epigenetic modification influenced by individual endogenous and exogenous environmental factors [18, 25, 26]; and reducing mutation rate caused by active demethylation [27] and X-chromosome inactivation [28, 29] (Fig. 1).

### Epigenomic reprogramming during later-life development

Fetal adaptation, an emerging concept in recent years, interprets the role of epigenetic regulation later in development, which is separated from early embryogenesis and implantation. In this context, subtle epigenetic modifications allow the fetal genotype to respond to a broad variety of developmental environmental factors. Although early gestation is the most susceptible period for the fetus, it should be noted that environmental stimulation in late embryonic development, infancy, and early childhood can also have long-term health implications in later life [9, 30]. Studies have shown that a high-fat diet (HFD) supplemented in adulthood induced large-scale methylation alteration in skeletal muscles [31]. Folic acid supplementation during the peri-pubertal period has been shown to induce hypermethylation of the *PPARα* gene and a decrease in DNMT activity [32, 33]. In addition, post-weaning mice supplemented with methyl donors-deficient diet showed a permanent loss of *IGF2* imprinting, dysregulation of mRNA expression, and hypomethylation of the proto-oncogenes such as *c-Myc* and *c-Ras* [34]. All these studies suggest that plasticity of the human epigenome may also persist into adulthood [31] and epigenetic mechanisms are involved in life-long adaptation [35].

### The roles of DNA methylation in gene expression and cellular identity

As one of the most important of the epigenetic modifications, DNA methylation can play a key role in local control of gene expression. CpG islands (CGIs) are short interspersed DNA sequences with a high frequency of CpG sites that are predominantly non-methylated [36]. A CGI is generally defined as a region with at least 200 bp and a CG percentage greater than 50%. The multiple methylated CpG sites in CGIs of promoters and distal regulatory regions may destabilize nucleosomes and recruit proteins, resulting in chromatin structure remodeling and transcriptional inhibition [37]. Methylated CpG sites can be recognized by different sets of methyl-CpG-binding proteins (MBPs), which then translate the DNA methylation signal into transcriptional repression states through attracting epigenetic modifiers to manage site-specific chromatin organization [38]. On the other hand, the methylation of CpG sites can block the binding of certain transcription factors, such as E2F1, c-Myc, CTCT, and CREB, obstructing transcription initiation [39]. DNA methylation can also reposition nucleosomes leading to remodeling transcription complexes and interrupting gene transcription. In addition, increasing evidence has indicated that gene expression may be simultaneously regulated by the methylation levels in the promoter region and the gene body [40, 41].

DNA methylation is also crucial and essential for the establishment and maintenance of cellular identity. Global hypomethylation is required for the pluripotency of embryonic stem cells (ESCs) [42]. During cell differentiation, ESCs gradually lose their plasticity and narrow their identity into differentiated cell types. In this process, there is a global gain of DNA methylation in pluripotency, developmental, and gamete-specific genes, along with the loss of DNA methylation in lineage-specific regulatory regions as well as gene enhancer regions, to define cell identities with different methylomic profiles [39]. As different tissues and organs have different methylomes, exposure to environmental factors may lead to altered DNA methylation patterns and adverse health outcomes in a tissue-specific manner [43–45].

### Epigenetic stability and environmental factors

Epigenetic stability is proportional to the amount of DNA methylation and histone modification in the static model [46]. Global hypomethylation of genomic DNA can lead to genomic instability and structural abnormalities in chromosomes, which is also a common phenotype of cancer and aging [47, 48]. Conversely, global hypermethylation, especially in the placenta, has been linked with developmental defects such as gestational diabetes and Down’s syndrome [49, 50]. Together, these show that the balance of DNA methylation is crucial for human genetic stability and individual health. In the dynamic model, epigenetic modification is reversible, thus making the epigenome persistently vulnerable. The proportion of stem cells contributes to epigenetic vulnerability of the organism, indicating that the gradual decline of overall epigenome stability with development may arise from, at least in part, the decrease of stem cell proportion in tissues and organs [46].

The epigenome, especially DNA methylation patterns in mammals including humans, is overall established in gametogenesis and early embryogenesis. The plasticity of the epigenome also contributes to the generation of cells with a broad developmental potential [18]. In this regard, epigenetic reprogramming in germ cells and the preimplantation embryo is particularly important for early embryonic and placental development [51]. This leads to a speculation that perturbations of the epigenome in early developmental stages contribute to abnormal fetal and placental development [52]. The epigenetic dysregulation triggered by environmental cues during these sensitive periods of individual development can persist across the life course leading to altered disease susceptibility and even phenotypic changes later in life [13, 14].

Studies have confirmed the developmental plasticity by which a specific genotype can give rise to a range of phenotypes in response to persistent environmental conditions during development [53–55]. DOHaD phenomenon also describes the relationship between early environmental cues and later-life risk of abnormal metabolism and other diseases, where epigenetic mechanisms could be the bridge connecting these factors [56–58]. The timing of an intervention is the key to epigenetic alteration in response to environmental pollutants such as endocrine-disrupting chemicals and heavy metal or bioactive food components. For instance, our recent studies showed that prenatal phytochemicals may affect epigenetic patterns more profoundly than the same exposure in postnatal or adulthood [59]. Likewise, the time windows of the intervention are particularly important for the efficacy of epigenetic perturbation to prevent individual abnormal development [60].

## Prenatal environmental pollution and epigenetic dysregulation

The concept of developmental programming emphasizes that during sensitive windows of vulnerability, environmental intervention may result in functional dysregulation of gene expression and disease pathogenesis in later life [61]. Early-life development, in particular during embryogenesis, has been shown to play an important role in the initiation and development of many chronic metabolic diseases as well as cancers, and epigenetic mechanisms have been suggested to be involved in these processes [35]. The general epigenome, including DNA methylation and histone modifications, is established in the early embryo and the germ cells and has been thought to maintain a very stable modification status throughout the life course. An expanding body of evidence has confirmed that environmental stimuli such as climatic factors and environmental toxicants, occurring especially during prenatal and early postnatal life, may alter epigenetic programming leading to altered disease susceptibility or irreversible phenotypic changes in the offspring [62]. Among these risk factors, prenatal exposure to environmental contaminants attracts more attention and has been repeatedly found to be associated with aberrant epigenetic modification of regulatory sequences in susceptible genes [63, 64]. Here, we review several prenatal environmental pollutants in different categories and their potential impacts on embryonic and postnatal development through epigenetic regulation.

### Ambient air pollution

Ambient air pollution includes particulate matter (PM) of various sizes and composition, as well as gaseous pollutants [65]. Early-life exposure to air pollution, especially during gestation, is a major health threat to pregnant women [66] and the developing fetus as well as the children. Air pollution has been shown to associate with various allergic complications both in short-term and long-term influence [67–69] as it can cross the placenta [15, 70, 71]. Although the specific molecular mechanisms underlying the effect of air pollution are not fully understood, epigenetic modifications are believed to be one of the key contributors that may link air pollution exposure to a range of adverse health outcomes [15, 72].

#### Particulate matter

Studies have shown PM with a diameter smaller than 500 nm can pass the placental barrier and particles even can reach the fetal bloodstream when their diameters are smaller than 240 nm [71]. Janssen et al. found that exposure to particles with aerodynamics diameter smaller than 2.5 μm (PM2.5), with 5 μg/m^3^, resulted in a decrease (2.2%) of global DNA methylation in placenta tissue [73]. It should be noted that altered placental global DNA methylation [73, 74] and gene-specific (*LINE1* and *HSD11B2*) methylation [75] were observed only when exposed to PM2.5 during early pregnancy, which includes the period from fertilization up to implantation and is most sensitive to environmental stress. Studies have also reported that prenatal exposure to PM was associated a decrease in placental mitochondrial DNA (mtDNA) contents [76] and DNA hypomethylation of the mitochondrial genome [77]. PM2.5 exposure has been shown to be linked with a decrease (0.2–2.7%, *P* < 0.05) of DNA methylation in the promoter region of the *leptin* gene, which is an important hormone during gestation and plays a key role in energy metabolism [78], as well as hypermethylation of the *PARP* promoter [79]. In addition, maternal exposure to particles also targets miRNAs. A decrease in expression of miR-21, miR-146a, and miR-222 was found to associate with PM2.5 exposure during the second trimester of pregnancy, whereas an increase in expression of miR-20a and miR-21 was observed during the first trimester [80] (Table 1).

**Table 1:** Summary of human studies reporting associations between prenatal exposure to air pollution and epigenetic alterations

Pollution	Exposure stage	Epigenetic change	Ref.
Particulate matter	Prenatal	Altered DNA methylation at CpG sites	[65]
	First trimester	Positive correlation with placental global DNA methylation	[74]
	Second trimester	Lower placental leptin promoter methylation	[78]
	Early pregnancy	Associated with placental DNA methylation of *LINE1* and *HSD11B2*	[75]
	Prenatal	Decreased expression of miR-21, miR-146a and miR-222; increased expression of miR-20a and miR-21	[80]
	Gestation	Increased mtDNA methylation levels and decreased *LINE-1* methylation levels	[77]
	Prenatal	Decrease in global DNA methylation for whole pregnancy	[73]
	Prenatal	Increased DNA methylation in *LINE1*, *OGG1*, *APEX* and *PARP1*	[79]
Smoking	Prenatal	Nearly 3000 CpGs corresponding to genes differentially methylated in offspring	[85]
	Maternal	Altered DNA methylation levels at CpG sites of *GFI1*, *AHRR* and *PRNP* gene in male and female, differentially	[110]
	In utero	Impact key biological pathways through epigenetic modification	[84]
	Maternal	Differential methylation of *MYO1G*, *CNTNAP2* and *FRMD4A* genes in children blood	[107]
	In utero	Global DNA hypomethylation; 31 CpG sites associated to 25 genes	[99]
	Prenatal	Altered methylation at 15 CpG sites	[100]
	Prenatal	Differential methylation at five CpGs in *MYO1G* and *CNTNAP2*; persist in exposed offspring for many years	[88]
	In utero	Increased CpG methylation in *FRMD4A* and *Cllorf52*; reproducible epigenetic changes persist into childhood	[87]
	In utero	Altered methylation at 185 CpGs of 110 gene regions in infants	[101]
	In utero	Hypomethylation of *AHRR* in the cord blood mononuclear cells, buccal epithelium and placenta tissue	[44]
	In utero	Altered methylation at *TSLP* promoter	[106]
	Maternal	Altered methylation patterns of a few loci within the *RUNX3* gene	[102]
	In utero	Increased *IGF2* DMR	[105]
	In utero	Altered *LINE-1* and *AluYb8* methylation levels	[83]
	Maternal	Differential DNA methylation at epigenome-wide for 26 CpGs mapped to 10 genes	[104]
	Maternal	Differential epigenome-wide placental DNA methylation	[82]
	Gestation	Decreased methylation of *Sat2*	[96]
	Maternal	Increased DNA methylation in the *BNDF-6* exon	[108]
	Gestation	Downregulation of miR-16, miR-21 and miR-146a in placenta	[109]
	In utero	Global DNA methylation inversely correlates with cotinine levels in cord blood	[95]
	In utero	Decreased methylation at *CYPIAI* promoter in the placenta	[103]
	Prenatal	Lower methylation of *AluYb8*; differential methylation of *LINE1*; increased methylation of *AXL* and *PTPRO*	[98]
Polycyclic aromatic hydrocarbons	Prenatal	Inverse relationship with *LINE1* DNA methylation in cord blood	[119]
	Prenatal	Decreased global methylation in umbilical cord white blood cells	[118]
	Prenatal	Altered methylation in 5′-CpG islands of *ACSL3*	[120]
NO_2_	Prenatal	Related alteration of *ADORA2B* methylation	[65]
	Prenatal	Differential offspring DNA methylation in antioxidant and mitochondria-related genes	[122]

#### Smoking

Maternal tobacco smoke is a personalized form of air pollution for the mother herself and fetus [73]. Although smoking is controllable, more than half of female smokers continue to smoke after pregnancy [81]. In utero exposure to smoking is associated with alterations of DNA methylation patterns [82–85], and such changes may persist throughout the entire life course [85–88], leading to impaired fetal development [83, 89], preterm delivery [90, 91] and other chronic diseases including respiratory illness, cardiovascular disorders, and certain childhood cancers in the offspring’s later life [92–94]. The methylation targets of maternal smoking during pregnancy could be genome-wide [85, 95–101] and specific such as runt-related transcription factor 3 (*RUNX3*), aryl hydrocarbon receptor repressor (*AHRR*), and cytochrome P450 1A1 (*CYP1A1*) in placental tissue [44, 102, 103]; *AHRR*, growth factor independent 1 (*GFI1*), insulin-like growth factor 2 (*IGF2*), PR domain containing 8 (*PRDM8*), discs large homolog-associated protein 2 (*DLGAP2*), thymic stromal lymphopoietin (*TSLP*), *CYP1A1* in newborn umbilical cord blood samples [44, 85, 104–106]; and Myosin 1 G (*MYO1G*), cortactin-associated protein-like 2 (*CNTNAP2*), and *FRMD4A*, a human epidermal stem cell marker, in children’s blood [107]; *MYO1G*, *CNTNAP2*, and brain-derived neurotrophic factor (*BDNF*) in adolescent’s peripheral blood cells [88, 108]; and repetitive element satellite 2 (*Sat2*) in adult peripheral blood granulocytes [96], as well as *AHRR*, in neonatal buccal epithelium [44]. Maternal tobacco smoking has been also linked to dysregulated expression of miRNAs. Maccani et al. [109] demonstrated that smoking during pregnancy downregulated the placental expression of miR-16, miR-21, and miR-146a that may influence fetal programming. Interestingly, the impact of prenatal cigarette smoke on DNA methylation may be gender-specific. It was shown that the male fetus is more susceptible to maternal smoking than the female [110], and the alteration of DNA methylation in the differentially methylated region (DMR) of the *IGF2* gene was more notable among newborn boys than girls [105], whereas Bouwland-Both reported an adverse result [111]. Moreover, a study has shown that maternal smoking showed a much stronger impact on offspring methylation intensity than paternal smoking [15] (Table 1).

#### Polycyclic aromatic hydrocarbons

Polycyclic aromatic hydrocarbons (PAHs) are aromatic hydrocarbons with two or more fused benzene rings [112]. They are mainly formed during incomplete combustion of fossil fuel, domestic wood, and other organic materials that are widely distributed in the air [113]. PAHs are persistent organic pollutants (POPs) that have detrimental biological effects such as genotoxicity and carcinogenicity [112, 114]. Some PAHs resemble steroid hormones and are lipid soluble, thereby resulting in accumulation in adipose tissue. These PAHs can even transfer across the placental barrier and the fetal blood-brain barrier. There is increasing evidence that prenatal exposure to PAHs results in multiple adverse effects on embryonic development [115–117]. In utero exposure to higher levels of PAHs has been associated with decreased genomic DNA methylation in American and Chinese cohorts [118, 119]. Prenatal exposure to traffic-related PAHs was also shown to be linked with hypermethylation of the acyl-CoA synthetase long-chain family member 3 (*ACSL3*) gene, which impacts asthma pathogenesis in umbilical cord blood of newborns [120] (Table 1).

#### Other air pollution

Pregnant women living near major roads may be easily affected by traffic-related air pollution and have been reported to show decreased DNA methylation in the long interspersed nuclear element 1 (*LINE-1*) gene in placenta tissue [121]. Aberrant DNA methylation patterns have been found in mitochondria-related and antioxidant defense-related genes of neonates who were prenatally exposed to NO_2_ [122]. In utero exposure to diesel exhaust has been associated with altered methylation of genes that are implicated in cardiovascular-related diseases and substance metabolism [123] (Table 1).

### Endocrine-disrupting chemicals

Endocrine-disrupting chemicals (EDCs) are a class of chemical compounds widespread in the environment [124]. EDCs are exogeneous synthetic or natural chemicals including phthalates (plastic-softening chemicals), polychlorinated biphenyls, pesticides, and dioxin class compounds, which exhibit hormone-like activity and can disrupt endocrine function by modifying, blocking, or mimicking the actions of endogenous hormones [15, 125–127]. There is increasing evidence that has suggested that prenatal exposure to certain EDCs can cause long-term health outcomes including cardiovascular disease, diabetes, infertility, and cancer [128–130]. Because the developing organism is extremely sensitive to hormone analogue [127]. These effects are also correlated with disruption in epigenetic programming [11, 131–133].

#### Bisphenol A

Bisphenol A (BPA) is an EDC of specific concern because of its high production and ubiquitous use in the manufacture of polycarbonate plastics in modern society [134]. The data have shown that BPA can be detected in 95% of human urine samples suggesting its widespread use or exposure [135]. Like particulate matter, BPA can also transfer across the placenta and accumulate in the fetus [136]. In utero exposure to BPA is associated with altered reproductive function, metabolic disorders and hormone-associated tumors such as breast and prostate cancer [137]. A study on mice showed that abnormal methylation patterns resulting from prenatally BPA exposure were established before germ layer differentiation in the embryonic stem cells [11], which may partially explain substantially adverse outcomes of BPA exposure [138–141]. Moreover, compelling evidence has been presented that offspring phenotype was also changed by stably altering the epigenome in a prenatally BPA-exposed mouse model [11]. Interestingly, altered miRNA expression was observed in in utero BPA-exposed sheep [142]. Gene-specific analysis of DNA methylation in humans found that altered methylation patterns of the placenta and the fetal liver and kidney were associated with prenatal exposure to BPA [114, 143, 144]. The genes catechol-*O*-methyltransferase (*COMT*) and sulfotransferase 2A1 (*SULT2A1*) are responsible for encoding two xenobiotic-metabolizing enzymes, and increased methylation at the promoters of these two genes has been revealed in BPA-exposed human fetal liver [145]. It is worth noting that perinatal exposure to environmentally relevant doses of BPA has also shown a transgenerational inheritance of male infertility through epigenome dysregulation in the male germ line [146–148] (Table 2).

**Table 2:** Summary of studies reporting associations between prenatal exposure to EDCs and epigenetic alterations

Chemical		Model	Exposure stage	Epigenetic change	Ref.
Bisphenol A		Mouse	In utero	Increased *EZH* gene expression in the mammary gland	[138]
		Human and mouse	Perinatal	Differential DNA methylation in repetitive DNA	[141]
		Human	In utero	Altered genome-wide DNA methylation in fetal liver	[144]
		Human	1st to 2nd trimester	Increased site-specific methylation at *COMT* and *SULT2A1* promoters in fetal liver	[145]
		Mouse	Preconception to weaning	Hypomethylation and increased expression of the *A*^*vy*^ gene in agouti mouse model	[11]
		Mouse	In utero	Decreased methylation in Hoxa10 gene promoter	[139]
		Rat	Perinatal	Modified hepatic DNA methylation	[140]
		Sheep	Prenatal	Altered microRNA expression	[142]
		Mouse	In utero	Both hyper- and hypomethylation at the promoter-associated CGIs	[132]
		Human	In utero	Positively associated with global methylation for the placenta	[143]
Vinclozolin		Mouse	In utero	Decreased methylation in *H19* and *Gtl2*; increased methylation in *Peg1*, *Snrpn*, and *Peg3*	[151]
		Rat	In utero	Altered epigenetic modification in the male germ line	[150]
		Rat	In utero	Altered methylation in sperm promoter epigenome of F3 generation	[152]
		Mouse	In utero	Epigenetic transgenerational inheritance of modifications in the mouse sperm epigenome	[124]
POPs	Dioxin	Mouse	In utero	Increased methylation in *Igf2r* gene in muscle and liver	[175]
		Mouse	Preimplantation	Altered methylation status of imprinted genes *H19* and *Igf2*	[176]
	Diethylstilbestrol	Mouse	In utero	Increased *EZH* gene expression in the mammary gland	[138]
		Mouse	In utero	Hypermethylation and long-term altered expression of the *Hoxa10* gene	[177]
		Mouse	Neonatal	Hypomethylation in Exon-4 of *c-fos*	[178]
	Methoxychlor	Mouse	In utero	Altered methylation in *H19* and *Gtl2* and increased methylation in *Peg1*, *Snrpn*, and *Peg3*	[151]
		Rat	In utero	Hypermethylation in the *ERβ* promoter regions	[179]
	PBDEs	Human	Prenatal	DNA hypomethylation of *Alu* and *LINE-1* in fetal blood	[168]
		Human	Maternal	Hypomethylation of *TNF-α* in core blood	[169]
		Human	In utero	Hypomethylation of *IGF2* and *NR3C1* in placenta	[170, 171]
	PFOAs	Human	Prenatal	Hypomethylation in sperm cells	[173, 174]
		Human	Prenatal	Global and *IGF2* hypomethylation in cord blood	[95, 172]

#### Vinclozolin

Vinclozolin is a systemic fungicide commonly used on fruit and vegetable planting and in the wine industry [149]. Researchers used vinclozolin as an EDC model to investigate epigenetic transgenerational inheritance of disease because of its anti-androgenic activity leading to spermatogenic defects, breast and prostate diseases, and even abnormal immune function at a high frequency (up to 90%) [1, 150–152]. Although female rat exposure to vinclozolin during gestation resulted in infertility in male offspring, the different timings of exposure may have different outcomes. Exposure during embryonic day (E) 8 to E 14, which is the period of germ line epigenetic programming, can reduce the spermatogenic capacity of male rats in four subsequent generations [131, 153], whereas vinclozolin exposure in later gestation (E 15–E 20) had no effect on fertility of adult males [154, 155]. Thus, exposure of male rats to vinclozolin in the early stage of embryogenesis can cause increased rates of infertility in adulthood and such effects can pass through four generations. Investigation of the molecular mechanisms of the aforementioned transgenerational phenomenon revealed that developmental exposure to vinclozolin substantially impacts reprogramming of the male germ line and induces aberrant methylation patterns which can be stably transmitted through multiple generations [156]. Differential DNA methylation identification in the F3 generation sperm epigenome could be used as epigenetic biomarkers for transgenerational influence assessments [124] (Table 2).

#### Persistent organic pollutants

Persistent organic pollutants (POPs) are a class of man-made organic (carbon-based) chemicals that remain for long periods of time after their introduction into the environment [157]. These chemicals include dichloro-diphenyl-trichloroethane (DDT), dichloro-diphenyl-dichloroethylene (DDE), polychlorinated biphenyls (PCBs), and 2,3,7,8-tetrachlorodibenzo-p-dioxin (TCDD), as well as perfluorooctanoic acid (PFOA), polybrominated diphenyl ethers (PBDEs), and dioxins [114, 157]. Certain POPs have been shown to have endocrine-disrupting effects such as estrogenic and anti-progestins of DDT, anti-estrogenic of dioxins and PCBs, anti-androgenic of DDT, and anti-thyroid of PCBs and dioxins (https://www.who.int/ceh/capacity/POPs.pdf). Accumulating evidence indicates that prenatal POPs exposures result in adverse mental and physical development [158–161], visual recognition memory abnormity [162], neurodevelopmental delay [163], reproductive problems [164, 165], obesity [166], and immune diseases [167] in the later life of offspring. Moreover, such adverse health effects from prenatal exposure to POPs are associated with epigenetic dysregulation, for instance, DNA hypomethylation of repeat elements (*Alu* (*Arthrobacter luteus*) and *LINE-1*) in fetal blood with exposure to DDT, DDE, and PBDEs [168]; hypomethylation of tumor necrosis factor alpha (*TNF-α*), *IGF2*, and nuclear receptor subfamily 3 group C member 1 (*NR3C1*) in core blood and placenta with exposure to PBDEs [169–171]; global and *IGF2* hypomethylation in sperm cells and cord blood samples with exposure to PFOA [95, 172–174]; altered DNA methylation in the *H19*, *IGF2,* and *IGF2r* genes with exposure to dioxin [175, 176]; hypermethylation of the *Hoxa10* gene, hypomethylation in the Exon-4 of *c-fos* gene, and increased *EZH* gene expression with exposure to diethylstilbestrol [138, 177, 178]; and increased methylation in the *Peg1*, *Snrpn*, *Peg3,* and *ERβ* genes with exposure to methoxychlor [151, 179]. In addition, certain POPs have been shown to promote epigenetic transgenerational inheritance of disease susceptibility [148, 180] (Table 2).

### Heavy metals

Heavy metals refer to metals with a density that exceeds a certain value (5 g/cm^3^) and have been used by humans in various areas for thousands of years [181]. Heavy metals including arsenic, cadmium, lead, and mercury are another common type of pollutant widely distributed in modern environments, such as various industrial, agricultural, medical, and domestic fields. The consumption of contaminated water or food is a common source of chronic, but low-level arsenic and cadmium exposure [182, 183]. Pesticide manufacturing is another common source of arsenic exposure [184], and smokers tend to have higher cadmium exposure [185]. Lead is often found in lead-contaminated house dust, residential soil, lead-based paints, glazed food containers, and drinking water [186, 187]. Contaminated seafood is considered the main source of mercury intake [188]. In utero exposure to heavy metals is detrimental for the fetus and mainly causes neurological disorders and cancers in the offspring [189]. Mounting evidence has revealed that such adverse outcomes are implicated with perturbation in the epigenome, which is susceptible to external stimulation during embryonic development [190] (Table 3).

**Table 3:** Summary of studies reporting associations between prenatal exposure to heavy metal and epigenetic alterations

Heavy metal	Exposure stage	Epigenetic change	Ref.
Arsenic	In utero	Altered DNA methylation status of specific genes in the placenta	[191]
	Prenatal	Altered DNA methylation in artery and placenta	[199]
	Prenatal	Altered DNA methylation in newborn cord blood	[192, 193, 196, 197]
	Early pregnancy	Decreased DNA methylation in cord blood	[194]
Cadmium	Prenatal	Differentially methylated CpG sites	[208]
	Early pregnancy	Altered DNA methylation at multiple DMRs in offspring with sex and possibly race/ethnic-specific effects	[203]
	Maternal	Decreased DNA methylation levels in placental *PCDHAC1* promoter	[206]
	Prenatal	Altered DNA methylation differently in girls and boys	[204]
	Maternal	Altered DNA methylation levels in the leukocyte of newborns and their mothers	[205]
Mercury	Prenatal	Increased DNA methylation in umbilical cord blood of infants	[190]
	In utero	Hypomethylation of *EMID2* gene in placental samples	[230]
	Prenatal	Related to DNA methylation at the *TCEANC2* region in cord blood samples	[231]
Lead	Prenatal	Hypermethylation at the *MEC3* DMR regulatory region	[223]
	In utero	Sex-specific trends between Pb and DNA methylation	[222, 227, 228]
	Prenatal	Hypomethylation of genomic DNA and *Alu* and *LINE-1* genes in cord blood	[224]
Manganese	Prenatal	Altered placental DNA methylation	[229]

#### Arsenic

Prenatal arsenic exposure has been shown to be associated with placenta and cord blood DNA methylation alteration in neonates [191–197], possibly in sex- [193, 194, 198] and time-specific [194] manners. For examples, the levels of DNA methylation were shown to increase in male infants but to decrease in female infants born to arsenic-exposed mothers [193, 198]. Arsenic exposure in late gestation showed a much weaker correlation with newborns cord blood DNA methylation than that in early pregnancy [194]. Furthermore, the effects of prenatal arsenic exposure on DNA methylation are not fully consistent in different studies. Some data supported negative correlation between arsenic exposure and methylation [194, 199], while certain studies demonstrated the role of arsenic in hypermethylation [199–201]. Collectively, these studies suggest that prenatal exposure to arsenic is believed to alter epigenetic modification and may dysregulate arsenic-related disease development Table 3.

#### Cadmium

Cadmium has a long half-life, lasting for decades, and can accumulate in the bones and then release during pregnancy. These features of cadmium magnify its toxicity to pregnant women and fetuses leading to numerous health problems such as reproductive disorders, kidney dysfunction, and certain cancers [202]. It was shown that early pregnancy exposure to cadmium leads to altered DNA methylation at multiple DMRs in offspring in sex- and possibly race/ethnic-specific manners [203]. Methylome-wide association study (MWAS) also demonstrated that prenatal, including periconceptional, and in utero exposure to cadmium resulted in increased methylation of organ development and mineralization-related genes in female offsprings, hypermethylation of cell death-linked genes in male offspring [204], and altered methylation patterns in leukocytes [205] and the placenta [206], as well as hypomethylation of *LINE-1*, which is hypermethylated in normal tissues [207] and peripheral blood. Another epigenome-wide association study of two US birth cohorts showed that prenatal cadmium exposure was associated with differentially methylated CpG sites, which were involved in inflammatory signaling and cell growth as well as birth weight [208]. Additionally, prericonceptional exposure to cadmium was found inversely associated with DNMT expression [207] (Table 3).

#### Lead

Lead is a common pollutant with no safe level of exposure and no beneficial biological role [209]. Likewise, lead accumulates in bone and has a half-life of about three decades [210]. Lead can elevate levels of homocysteine, disrupt the methionine-homocysteine cycle [211, 212], and reprogram the expression of epigenetic modification-related enzymes [213]. Together, these processes, exposure to lead, especially prenatally, may cause aberrant DNA methylation [214–216] and histome modifications such as histone acetylation [217] in organisms. Such alterations in the epigenome are likely preserved at first [218, 219], and then triggered by internal and/or external stimulation in later life resulting in clinical abnormalities such as obesity, cardiometabolic disease, and even Alzheimer’s disease (AD) [220–223]. Children who were prenatally exposed to lead displayed hypomethylation of *Alu* and *LINE-1* sequences [224], as well as altered methylation patterns in imprinted genes [222, 223]. Moreover, a study on animals showed that lead exposure can also alter the expression of miRNAs which target certain proteins participating in the pathologic process of disease [225], while no effect was found when exposure occurred in later life. Importantly, maternal lead exposure may leave a methylome fingerprint on her grandchildren, suggesting its potential multigenerational epigenetic inheritance [226]. Moreover, pronounced sex-specific profiles to prenatal lead exposure were also found with respect to DNA methylation alterations [222, 227, 228] (Table 3).

#### Other heavy metals

In utero exposure to manganese has been associated with differential methylation in the placenta [229]. DNA methylation changes, which were linked with altered immune profiles or adverse infant neurobehavioral outcomes, were found in the placenta as well as umbilical cord blood in newborns whose mothers had experienced mercury exposure during pregnancy [190, 230, 231] (Table 3).

### The characteristics of prenatal exposure-related epigenetic dysregulation

#### The portal function of the placenta

Exposure-related alterations in fetal development result in potential changes in metabolism and development [232]. As a transient organ, the placenta serves as a gatekeeper between the fetal and maternal circulation throughout pregnancy, ensuring survival of the fetus [61, 73]. It not only plays crucial roles in mediating the transfer of oxygen, nutrient substance, and hormones from mother to fetus [233], but also can produce growth factors and hormones and mediate fetal immune tolerance [61]. Adverse environmental factors during embryonic development may disrupt all placental functions of transportation, metabolism, protection, and endocrine, and such effects can be encoded in the placental methylome [234, 235], which will provide a unique footprint of exposures [65]. Hence, the placenta exhibits considerable plasticity, especially a distinctive DNA methylome [232, 236, 237]. However, if placental capacity to adapt is exceeded, fetal growth and development may be compromised directly [61]. Moreover, certain environmental toxicants can cross the placenta causing distorted fetal reprogramming and disease pathogenesis in later life [238].

#### Transgenerational inheritance

Transgenerational inheritance is often used rather broadly to describe non-DNA sequence-based inheritance that can be transmitted from one generation of an organism to the next [239, 240]. The F3 generation (the offspring of the F2 generation) is the first generation that exhibits transgenerational inheritance as both the F1 (the offspring of the parent generation) embryo and the F2 (the offspring of the F1 generation) germline involve direct exposure when an F0 (the parent generation) gestating female is exposed to an environmental factor [241–244]. Of great concern is that prenatal environmental exposure-induced epigenetic modifications may pass across subsequent generations through the germ line, leading to predisposition to diseases or disorders in the offspring [1, 30, 245]. Guerrero-Bosagna et al. proposed plausible molecular mechanisms/conditions for environment-induced epigenetic transgenerational inheritance including stepwise processes: first, exposure during gametogenesis; second, epigenetic insults in PGCs; third, imprinting-like programming in the germ line, especially in the male germ line, escaping reprogramming during early embryonic development; fourth, altered epigenome in the germ line transmitted to subsequent generations in cells and tissues; and finally, increased susceptibility to related diseases in postnatal life [124]. Epimutations mainly on DNA methylation resulting from F0 generation gestating female exposure to EDCs have previously demonstrated transgenerational inheritance through the male germ line [242, 246, 247]. It should be pointed out that sperm epimutations can magnify with increasing passages [242].

#### Time/age-specific susceptibilities

The distinct time windows, i.e., preconception, early gestation, infancy, and old age, are characterized by age-specific disease susceptibility [248]. As the epigenome is undergoing dynamic change and is vulnerable, the periods of early fetal development and the gamete formation are thought to be most susceptible to environmental stimulations. Human pregnancy has three trimesters: trimester 1, from 1 to 13 weeks; trimester 2, from 14 to 26 weeks; and trimester 3, from 27 weeks to delivery. Thus, the first trimester from fertilization to implantation undergoing epigenetic reprogramming that is highly sensitive to environmental stimuli is considered the most important developmental stage and can decide later-life disease susceptibility in the offspring.

#### Sex-specific response/profile

Dynamic processes of epigenetic reprogramming in male and female genomes exhibit dramatic differences [14, 20] and this includes changes to the epigenome in their embryonic stem cells [249]. As aforementioned, male fetus has been observed a higher susceptibility to maternal smoking than the female [110]. Developmental exposure to vinclozolin [156] and BPA [146–148] has been shown a transgenerational inheritance of aberrant methylation patterns through the male germ line. Moreover, early pregnancy exposure to cadmium [203, 204] and lead [222, 227, 228] resulted in altered DNA methylation in offspring in a sex-specific manner.

### The potential mechanisms of prenatal exposure-related epigenetic dysregulation

#### Oxidative stress

**Fig. 2:**
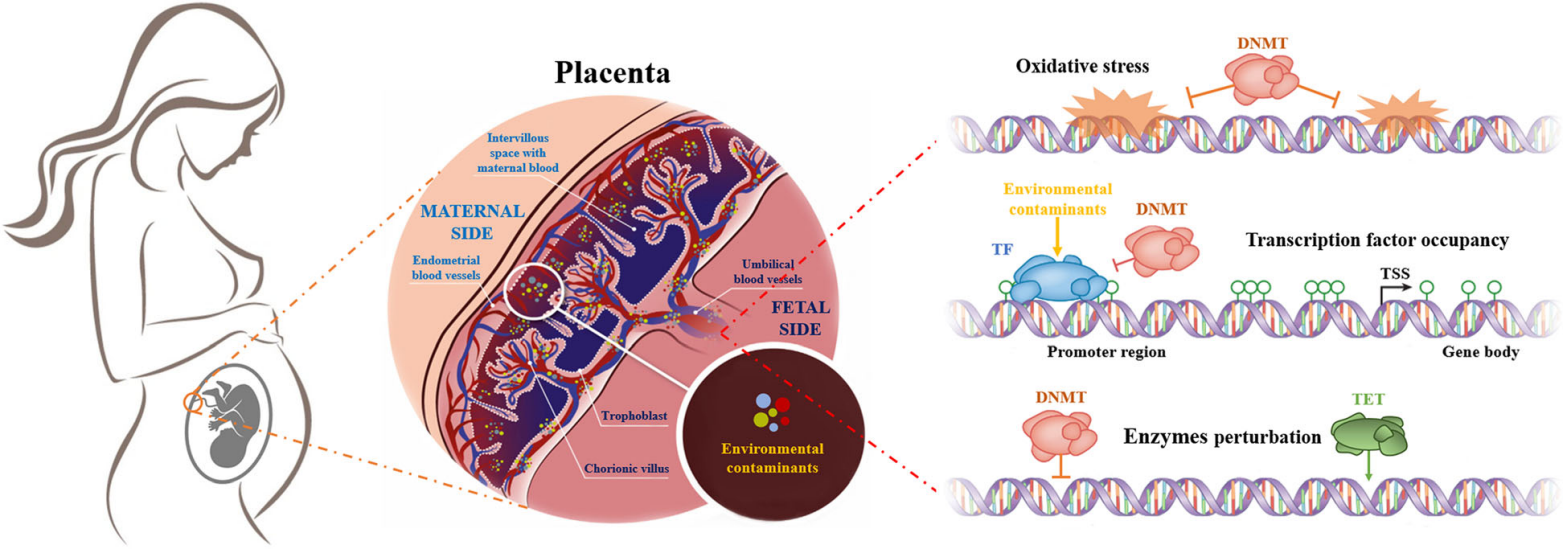
Diagram of the potential mechanisms of contaminant-induced epigenetic dysregulation. Environmental contaminants can be absorbed into the maternal blood through breathing, ingestion, drinking, or contact. Subsequently, certain environmental toxicants can pass the placental barrier and accumulate in the fetal bloodstream, causing epigenetic dysregulation through three potential ways: DNA oxidative damage may block the binding of DNA methyltransferase (DNMT) to the DNA template; activation of transcription factors (TFs) can inhibit DNMTs from accessing the DNA, resulting in gene-specific hypomethylation; interfering activity of DNMT or/and ten-eleven translocation (TET) enzyme families, leading to genomic methylation dysregulation. Adapted and used with permission from Martin et al. [114] and Luyten et al. [232]. Abbreviations: DNMT, DNA methyltransferase; TSS, transcription start site; TF, transcription factor; TET, ten-eleven translocation

Taking PM as an example, inhaled particles may first translocate from the maternal lung into the bloodstream, then pass through the placental barrier and induce oxidative stress [122, 250, 251]. DNA damage induced by oxidative stress has been associated with differential methylation in several candidate genes in response to prenatal exposures [120, 252]. DNA damage may block the binding of DNMTs, whose dysfunction is lethal to developing embryos [253], to the DNA template thereby causing hypomethylation [254]. Well-documented evidence demonstrates that DNA hypomethylation can induce genomic and chromosomal instability [255–257], and has been linked with abnormal embryonic development [258] such as spina bifida [259] and low birth weight [260] of newborns (Fig. 2).

#### Transcription factor occupancy

For gene-specific DNA methylation patterns, the transcription factor occupancy theory proposes that the blocking approach to DNA methylation machinery may occur due to the presence of transcription factors on gene regulatory region, or vice versa [261, 262]. In a similar manner, if environmental chemicals trigger the presence or absence of transcription factors on a gene regulatory region, this may result in site-specific methylation patterns [114] (Fig. 2).

#### The perturbation of related enzymes

For genome-wide patterns of methylation, it has been shown that environmental cues may change the function of DNMT or/and ten-eleven translocation (TET) enzyme families as well as the availability of *S*-adenosylmethionine (SAM) to DNA [114] leading to genomic hypomethylation or hypermethylation (Fig. 2).

Obviously, epigenetic modifications are potentially reversible, and a deeper understanding of the characteristics and mechanisms implicated in adverse outcomes of prenatal environmental stimulations will likely promote exploration of new effective therapeutic methods targeting anomalous epigenetic markers. Until the present, some histone deacetylase (HDAC) inhibitors and DNMT inhibitors, which are approved by FDA, have been used in epigenetic pharmacological therapies, providing clinical benefits through inhibiting HDACs or DNMTs [15]. Nevertheless, recent studies elucidate that certain bioactive compounds in “epigenetics diets” may act as DNMT inhibitors, HDAC inhibitors, or/and miRNA regulators that influence the epigenetic profile and play a potential protective role against environmental pollution.

## Epigenetics diets and their roles on epigenetic regulation

Early-life dietary nutrition can profoundly affect developmental fate through the altered epigenome [35]. Female larvae can develop into queen bees or sterile worker bees in the presence or absence of royal jelly, which is the most typical example of nutrition epigenetics [263]. However, the proportion of larvae developing into queen bees would increase with the knockdown of DNMT3, suggesting the bridge role, at least in part, through DNA methylation between early-life royal jelly consumption and adult phenotype [264]. Early-life supplementation of certain foods can also have detrimental effects on the developing fetus. Results from a meta-analysis showed that prenatal alcohol exposure may disturb protein synthesis, mRNA splicing, and chromatin regulation in rodent embryos [265]. Of great interest is that a number of bioactive dietary components act to modify the epigenome through consumption of so called “epigenetics diets” [30]. Here, we detail epigenetic diets and their roles in epigenome modification (Table 4).

**Table 4:** Epigenetic diets and their properties in epigenetic regulation

Classification	Food example	Component	Epigenetic effect	Ref.
Polyphenol		Kaempferol	HDAC inhibition	[323, 324]
			SIRT3 activation	[322]
		Phloretin	DNMT inhibition	[325]
		Apigenin	DNMT inhibition	[326, 327]
			HDAC inhibition	[326]
			HMT inhibition	[327]
		Luteolin	DNMT inhibition	[327, 328, 330]
			HDAC inhibition	[329, 330]
			HMT inhibition	[327]
			SIRT activation	[329]
		Hesperidin	DNMT inhibition	[331]
		Quercetin	DNMT inhibition	[275, 326, 332]
			HAT inhibition	[334]
			SITR1 activation	[333, 344]
		Caffeic acid	HDAC inhibition	[335, 336]
		Chlorogenic acid	HDAC inhibition	[335]
		Allyl mercaptan	HDAC inhibition	[337]
		Diallyl disulfide	HDAC inhibition	[338–340]
		Anthocyanin	DNMT inhibition	[325, 341]
			miRNAs modulation	[342]
		Piceatannol	SIRT1 activation	[333, 343, 344]
		Procyanidin	DNMT inhibition	[283, 479]
			HDAC inhibition	[283, 480]
			SIRT1 modulation	[346, 347]
			miRNA modulation	[342, 345, 348]
		Resveratrol	DNMT inhibition	[268, 283–286]
			HDAC inhibition	[283, 285, 287–289]
			miRNAs modulation	[284, 299–303]
			SIRT1 activation	[292–296]
			Decreased MeCP2	[285]
		Catechin (EGCG)	DNMT inhibition	[274, 275, 285, 481]
			HAT inhibition	[270, 482]
			HDAC inhibition	[276, 285, 481, 483]
			Decreased MeCP2	[285]
			miRNAs modulation	[278, 279, 281]
		Theophylline	HDAC activation	[349, 350]
		Biochanin A	DNMT inhibition	[350]
		Daidzein	DNMT inhibition	[351]
		Equol	Demethylation of BRCAs	[352]
		Genistein	DNMT inhibition	[285, 306–309]
			Decreased MeCP2	[285]
			HDAC inhibition	[285, 306, 309]
			HAT activation	[311, 313]
			miRNAs modulation	[271, 318–321]
		Curcumin	DNMT inhibition	[285, 353, 484, 485]
			Decreased MeCP2	[285]
			HAT inhibition	[354]
			HDAC inhibition	[285, 355, 356]
			miRNAs modulation	[357, 486–489]
Vitamin		Folate	One-carbon metabolism	[386, 490, 491]
			HMT regulation	[491]
			Epigenome regulation	[492]
			miRNAs modulation	[405, 493–495]
		Vitamin C	DNA demethylation	[360–366]
			Histone demethylation	[367–370]
			Epigenome regulation	[358]
		Vitamin D	DNA methylation	[375–379, 468, 469, 496]
			Histone modification	[372, 374, 380, 381]
			Epigenome regulation	[372, 470]
			miRNAs modulation	[497–499]
		Choline	DNA methylation	[393–398, 401–404]
			Histone methylation	[405]
			miRNAs modulation	[405–407]
Other		Isothiocyanate	HDAC inhibition	[419–422]
		Sulforaphane	HDAC inhibition	[269, 276, 409–412]
			DNMT inhibition	[276, 414, 415]
			miRNAs modulation	[414, 416–418]
		Withaferin A	DNMT inhibition	[285, 411]
			HDAC inhibition	[269, 285, 411]
			HMT inhibition	[269]
			HAT activation	[269]
			miRNAs modulation	[500]
		Se	DNA methylation	[436, 437, 439–444]
			Histone modification	[436, 438]
			DNMT inhibition	[436]
			HDAC inhibition	[438]

### Polyphenols

Polyphenols are widely distributed secondary metabolites from plant origin, especially fruits and vegetables [266]. Accumulating literature indicates that these phytochemicals have antioxidant, anti-inflammatory, and other beneficial effects on human health [267]. Many polyphenols have shown properties in regulation of epigenetics, such as DNMT inhibition by resveratrol in grapes [268], HDAC inhibition by sulforaphane in broccoli [269], histone acetyltransferase (HAT) inhibition by (−)-epigallocatechin-3-gallate (EGCG) in green tea [270] as well as miRNA regulation by genistein in soybean [271].

#### EGCG

Catechins are the most abundant polyphenolic compounds in green tea, among which EGCG accounts for more than 50% of the active compounds [272, 273]. Apart from its known roles in DNA methylation [274–276], EGCG also acts as a histone modifier and miRNA modulator. Compared with other green tea polyphenols, EGCG exhibits the most potent HAT inhibitor properties targeting various HAT enzymes including p300, CBP, Tip60, and PCAF [270]. Our study demonstrated that combined with SFN, EGCG can remodel chromatin structure by histone modification as well as change methylation patterns in the *ERα* promoter, thereby reactivating *ERα* expression and then re-sensitizing anti-hormone (tamoxifen) treatment in ER-negative breast cancer [276]. In another study, EGCG has shown to affect Polycomb-group (PcG) proteins which can compact chromatin and silence cancer-related genes through regulating histone methylation and acetylation [277]. Additionally, EGCG has also been found to modulate miRNA expression in human nasopharyngeal carcinoma CNE2 cells [278], osteoarthritis chondrocytes [279], osteosarcoma cells [280], and spontaneously hypertensive rat [281] (Table 4).

#### Resveratrol

Resveratrol (RSV) is a natural polyphenolic compound and is often found in peanuts, berries, and grape species, especially in the skin of red grapes [282]. RSV exhibits antioxidant, anti-inflammatory, antiangiogenic, and anticancer properties through epigenetic regulations via its abilities of DNMT [268, 283–286] and HDAC inhibition [283, 285, 287–289]. Sirtuin 1(SIRT1) is a NAD+-dependent histone deacetylase which deacetylates proteins that contribute to oxidative stress, aging, obesity, and tumors [290]. Importantly, SIRT1 is also involved in the regulation of DNMT1 activity [291]. A body of investigations indicates that RSV is associated with SIRT1 activation in various metabolic pathways [292–298]. Moreover, new studies suggest that RSV acts as a miRNA regulator in thrombus resolution [299], type 2 diabetes (T2D) treatment [300], clinical pancreatic ductal adenocarcinoma (PDAC) prevention [301], osteoarthritis therapy [302], and anti-inflammation [303] (Table 4).

#### Genistein

Genistein (GE) is a phytoestrogen and the major isoflavone primarily present in soy [304]. GE has been shown to exhibit health beneficial properties including inhibition of obesity, insulin resistance, and metabolic diseases, preventing inflammation and multiple cancers [305]. As aforementioned, polyphenols such as GE also exhibit striking effects on DNA methylation [285, 306–309] and histone modification [285, 306, 309, 310]. It was shown that some tumor suppressor-related genes, such as *p16*, *p21*, *RARβ*, *CCND2*, *GSTP1*, *MGMT*, and *BTG3*, were reactivated by GE-mediated promoter hypomethylation or/and histone hyperacetylation [311–316]. In our preliminary study, GE was also found to repress human telomerase reverse transcriptase (*hTERT*), which is the catalytic subunit of human telomerase, by locus-specific hypomethylation as well as chromatin structure remodeling of the *hTERT* promoter in breast cancer models [317]. Furthermore, GE may act as a miRNA modulator in breast, prostate, colorectal, and renal cancer prevention [271, 318–321] (Table 4).

#### Other polyphenols

Other polyphenols are also implicated in various health beneficial effects in human and animals through, at least in part, their properties in DNA inhibition, HDAC inhibition, HAT activation, and miRNA modulation such as kaempferol [322–324] and phloretin [325] in apple; apigenin [326, 327] and luteolin [327–330] in celery; hesperidin [331] and quercetin [332–334] in citrus; caffeic acid [335, 336] and chlorogenic acid [335] in coffee; allyl mercaptan [337] and diallyl disulfide [338–340] in garlic; anthocyanin [325, 341, 342], piceatannol [333, 343, 344], and procyanidin [283, 342, 345–348] in grape; theophylline [349, 350] in green tea; biochanin A [350], daidzein [351], and equol [352] in soy; and curcumin in turmeric [353–357] (Table 4).

### Vitamins

#### Vitamin C

Vitamin C (L-ascorbic acid) is known for its essential role in collagen crosslinking [358]; thus, its severe deficiency may cause scurvy [359]. Recent investigations have revealed functions of vitamin C in epigenetic regulations. Ascorbate, the form of vitamin C existing under physiological pH conditions, is found to be involved in active DNA demethylation [360–366] and histone demethylation [360, 367–369] as well as epigenome reprogramming [358] in a cofactor manner. TET dioxygenase, catalyzing the oxidation of 5mC into 5-carboxylcytosine (5caC) that are ultimately replaced by unmodified cytosine, has three cofactors, among which ascorbate is recently discovered and verified. The Jumonji C (JmjC)-domain-containing histone demethylases (JHDMs) including JHDM1A, 1B, and 3A also need ascorbate as a cofactor for histone demethylation [369–371]. Furthermore, a recent study revealed a specific role for vitamin C in H3K9me2 demethylation in mouse embryonic stem cells [368] (Table 4).

#### Vitamin D

The discovery of the calcitriol receptor, commonly known as the vitamin D receptor (VDR), gradually uncovers the roles of vitamin D in regulating transcriptional responses and underlying epigenetic mechanisms [372]. VDR is a member of transcription factors. The active form of vitamin D can bind to calcitriol [373], while VDR mainly binds at loci of open chromatin. Upon treating human leukemia cell lines, THP-1, with 1,25-dihydroxyvitamin D_3_ (1,25-D3), a VDR ligand, chromatin accessibility substantially increased [374]. Primary roles of vitamin D on epigenetic regulation are associated with DNA demethylation and histone acetylation. There is evidence showing that vitamin D treatment is negatively correlated with promoter methylation status of the adenomatous polyposis (*APC*) gene, a tumor suppressor gene in colorectal cancer [375], as well as dickkopf-related protein 1 (*DKK1*) [376], E-cadherin [377], PDZ-LIM domain-containing protein 2 (PDLIM2) [378] and *p21* [379]. In in vitro experiments, 1,25-D3 treatments have been shown to regulate gene expression through histone acetylation and methylation, such as H3K27ac [374], H3K9 di-methylation [380], and H3K9ac [381], as well as affecting the expression of a series of JHNMs [372]. Recent studies have revealed vitamin D anticancer properties through miRNA modulation (reviewed in [382]) (Table 4).

#### Folate

Folate or folic acid, also known as pteroylglutamic acid, is a water-soluble B-complex vitamin and usually exists in green vegetables and animal liver. Biologically, folate together with vitamin B12 (VB12) plays a crucial role in the one-carbon metabolism and embryonic development. In this context, low dietary intakes of folate are associated with various clinical symptoms, especially neurological and developmental disorders [383]. As a methyl donor, folate takes part in the methionine cycle and ultimately offers methyl for DNA and protein methylation, thereby changing chromatin structure and modulating gene expression [384]. Although DNA hypomethylation resulting from poor folate status is linked with inappropriate expression of cancer-related genes [385], it should be pointed that folate depletion can cause both hypo- and hypermethylation of DNA [386]. Furthermore, folic acid supplementation has been shown to reduce the risk of cancer [387, 388] through regulation of DNA methylation patterns [389, 390] (Table 4).

#### Choline

Like folate, choline is one of the precursors that can be converted to SAM, the universal methyl donor for numerous methylation processes including the methylation of cytosine in DNA, lysine in histones, and adenine in RNA as well as other molecules [391, 392]. Feeding pregnant methylation-indicator-mice a diet high in choline and other methyl donors resulted in offspring born with a brown coat and kinks in their tails through altering methylation status of *A*^*vy*^ [393, 394] and *Axin* (Fu) [395] genes, respectively. Several other examples have also demonstrated that dietary supplementation with choline changed methylation levels of CpG sites in the genes *IGF2*, *Srebf2*, *Agpat3*, *Esr1*, *Fasn*, and *Cdkn3* [396–398]. On the other hand, upon treatment of pregnant rats with choline-deficient diets, *IGF2* was hypermethylated through upregulating *DNMT1* expression [399]. In humans, the maternal supply of choline is essential for fetal and infant development, especially for brain development. Thus, extra choline is needed for pregnant and breast-fed women. Additionally, choline has a role in reducing human tumor progression. As evidence of this, Sun et al. found that low choline intake increases overall risk for lung cancer (30%), nasopharyngeal cancer (58%), and breast cancer (60%), whereas cancer incidence reduces by 11% after choline (100 mg/day) supplementation [400]. Studies have been well documented that choline can inhibit cancer development via modifying epigenetic markers. Choline-deficient diets result in hypomethylation of oncogene (e.g., *c*-*myc*) [401], but also hypermethylation of several tumor suppressor genes (e.g., *p16*, *p53*, and *Cx26*) [402–404]. Moreover, dietary choline concentration also affects histone methylation [405] and miRNA expression [405–407] (Table 4).

### Other epigenetics diets

#### Isothiocyanates

Isothiocyanates (ITCs) are generated by the enzymic hydrolysis of glucosinolates in plants. Sulforaphane (SFN) is an isothiocyanate that is present naturally in cruciferous vegetables such as broccoli, kale, cabbage, radish, and mustard [30, 408]. Increasing interest has focused on SFN-mediated chemoprevention due to its proven potent activity in HDAC inhibition [269, 409–412], which may lead to increased histone acetylation genome-wide as well as at specific-gene levels as histone acetylation is unequivocally linked with increased propensity for gene transcription [413]. Moreover, SFN has been shown to have properties in DNMT inhibition [276, 414, 415] and miRNA modulation [414, 416–418]. Except SFN, other ITCs [419–422] have also been shown various health beneficial effects in human and animals through their properties in epigenetic modification (Table 4).

#### Withaferin A

Withaferin A (WA), the first described withanolide, is a natural steroid lactone derived from *Withania somnifera* and has been attracting increasing interest because of its multifunctional properties including anti-inflammatory [423, 424], antimetastatic [425], anti-angiogenesis [426], and especially antitumor activity [427–429]. Importantly, WA exerts strong anticancer activity in mammary tumors at pharmacologically achievable concentrations [430]. In a recent study conducted by vel Szic et al., both triple-negative MDA-MB-231 and estrogen receptor-positive MCF-7 cells showed global DNA hypermethylation once treated with WA, and DNA methylation levels in MDA-MB-231 were lower than MCF-7 cells. Meanwhile, methylation perturbation-related specific genes were bidirectional (both hyper- and hypomethylated) and were contrary between these two cell lines. The authors also found that the observed hypermethylation has been linked with decreased H3K4me3 at the *PLAU* gene promoter [431]. In an earlier in vitro study, however, cells treated with 8 and 10 μM WA exerted DNMT inhibition activity [285]. In addition, WA has been associated with a decreased chromatin accessibility at the *IL-6* gene promoter region [432]. Two studies from our lab also illustrated WA acted as DNMT and HDAC inhibitors in breast cancer cells, and such activities were strengthened once combined with SFN [269, 411] (Table 4).

#### Selenium

Selenium (Se) is an essential trace element usually found in cereals, nuts, and vegetables [433], and has different forms including selenocysteine, sodium selenite, and sodium selenide [434]. Se has been received considerable attention for its beneficial effects toward human health such as immunity enhancement and anticarcinogenic action. Adequate selenium intake during pregnancy can also promote successful and healthy pregnancies through protecting against oxidative stress [435]. Nevertheless, mounting investigations have linked its priorities in regulation of epigenetic mechanisms, especially DNA methylation. Treated prostate cancer cells with Se have been shown to reactivate the expression of *GSTP1* by upregulating partial promoter DNA methylation levels and H3K9ac, while inhibiting HDAC activity as well as H3K9 methylation [436]. In addition, Se deficiency resulted in genomic DNA hypomethylation and promoter hypermethylation of *p16* and *p53* [437]. Furthermore, Miranda et al. found that sodium selenite and methylseleninic acid both can inhibit *DNMT1* expression in breast cancer cells. In addition, decreased H3K9me3 and H4K16ac were observed in methylseleninic acid and sodium selenite treated groups, respectively [438]. In mouse and rat studies, diet supplemented with Se resulted in increased DNA methylation in colon tissue [439, 440] and decreased global DNA methylation in liver [441, 442] and in heart [443], as well as increased methylation in the exon-specific locus of *Tp53* [442] and promoter regions of two inflammatory-related genes (*TLR2* and *ICAM1*) [444] (Table 4).

A growing body of evidence shows that dietary nutritious and non-nutritious components of vegetables, fruits, nuts, and beverages can regulate epigenetic processes (e.g., covalent modification of DNA, protein and RNA, miRNA modulation, chromatin remodeling) involved in critical life processes of human health such as immune improvement, apoptosis inhibition, and cancer prevention (Table 4). Their potential protective roles against environmental pollution have been attracting increasing attention.

## The potential protective roles of prenatal epigenetics diets against environmental pollutants

It is now clear that prenatal exposure to environmental pollutions induces adverse outcomes of embryonic and postnatal development through epigenetic dysregulation. In a similar manner, parental nutritional exposure may also induce long-term epigenetic perturbation in the offspring, determining the health of descendants throughout lifetime [30, 445, 446]. The former often occurs in a passive situation and leads to severe health issues in humans, whereas nutritional intervention is controllable and often beneficial. Increasing numbers of studies have shown potential properties of dietary compounds in epigenetic pharmacological therapies and chemoprevention. As a typical example, studies carried out by Dolinoy et al. demonstrated that a maternal methyl diet and phytoestrogen supplementation counteracted coat color change and hypomethylation in offspring induced by in utero and neonatal exposure to BPA [11], suggesting that maternal nutritional supplementation could be a potential preventive approach to attenuate or negate epigenome dysregulation resulting from environment stimulation. Here, we review the potential possibilities of prenatal nutrition against environmental exposure via epigenetic regulation.

### Maternal diets vs. EDCs

As noted above, BPA is a typical, ubiquitous endocrine-active compound. SAM functions as a universal methyl donor for methylation processes in DNA, protein, and RNA. B vitamins including folic acid, VB6, and VB12, as well as amino acids, such as choline, methionine, and betaine, are classified as methyl donor nutrients as they all either directly or indirectly act as precursors of SAM. In Dolinoy’s study [11], they first exposed female mice to 50 mg/kg BPA diet 2 weeks before mating with Avy/a males and throughout gestation and lactation. A changed coat color was found to be associated with decreased methylation of nine CpG sites of the *Agouti* gene. Strikingly, BPA-induced DNA hypomethylation in the offspring was negated after female mice were supplemented with methyl donors in their diet (4.3 mg of folic acid/kg diet, 0.53 mg of vitamin B12/kg diet, 5 g of betaine/kg diet, 7.97 g of choline chloride/kg diet). Although it is not clear which nutrients specifically played a more critical role in this mixed methyl diet, elevated methylation may reverse hypomethylation on the epigenome caused by EDC, indicating paternal methyl donor supplementation could be a potential nutrition intervention against prenatal EDC exposure. Importantly, shifted coat color distribution brought by a maternal methyl donor diet through hypermethylating-related genes in A^vy^ offspring was shown to be inherited through multiple generations [447], suggesting nutrient-reversed BPA-induced epigenome alterations can be transmitted transgenerationally through epigenetic inheritance via germline transmission [146–148]. In addition, dietary vitamin B supplementation appears to attenuate the adverse effects caused by pesticides in paint [448].

Maternal dietary exposure to genistein, which is a plant phytoestrogen primarily present in soy, also has been shown to shift offspring coat color by upregulating genomic methylation [449]. In Dolinoy’s study, upon treating virgin a/a female mice with 50 mg/kg diet of BPA and 250 mg/kg diet of genistein, BPA-induced hypomethylation in the *Agouti* gene of offspring was neutralized [11]. As polycarbonate plastics, like BPA, are ubiquitously used in the human population, and soybean products are widely consumed, the ability of genistein to prevent negative environmental toxicant effects via prenatally nutritional intervention has a promising prospect.

### Maternal diets vs. smoking

DNA methylation markers could be potential indicators of paternal smoking as methylation alteration of a series of genes has been shown to link to cigarette use. Among these genes, hypomethylation of *AHRR*, particularly at cg05575921 loci, was often found [104, 450–452]. In a recent study on African-American cohorts, smoking-induced DNA demethylation at *AHRR* was moderated by increased methylation of methylene tetrahydrofolate reductase (*MTHFR*), which is a key regulator in methyl metabolism [453]. Consistently, Zhang et al. found that sufficient maternal folate level could partly mitigate the adverse effect of maternal smoking on the epigenome of newborns, as well as on child health [110]. Moreover, Richmond and Joubert contrasted the effects of maternal smoking and one-carbon micronutrient exposures on the DNA methylome in the offspring and found that these two categories of exposure have potential opposite impact on the offspring epigenome and act independently [454].

### Maternal diets vs. metabolic syndrome

Metabolic syndrome (MetS) is a progressive phenotype that is characterized by a series of metabolic disorders such as obesity, hypertension, dyslipidemia, and insulin resistance [30, 455]. As reviewed above, maternal exposure to environmental pollutants has been shown to result in MetS with similar epigenome dysregulation in offspring. It was shown that maternal dietary methyl donors may regulate MetS through epigenetic mechanisms. Wolff et al. revealed that methyl donors supplementation in pregnant A^vy^/a mice prevented MetS phenotypes in offspring by DNA hypermethylation [393]. In addition, a methyl diet (folate, VB12, betaine, and choline) has been shown to prevent obesity in the same mouse strain [456] through DNA hypermethylation. Similar studies in humans also demonstrated that prenatal folic acid supplementation can reduce MetS incidence in children in rural Nepal [457], while disproportionality of folate and VB12 during gestation leads to insulin resistance and obesity in the offspring [458].

Maternal soybean supplementation also induced locus-specific DNA hypermethylation in A^vy^ intracisternal A particle (*IAP*) retrotransposon of heterozygous viable yellow agouti (Avy/a) offspring, shifting their coat color toward pseudoagouti, meanwhile decreasing obesity incidence in adulthood [449].

### Diets vs. ambient fine particles

Exposure to PM may induce systemic inflammation and oxidative stress through epigenome dysregulation. In a recent striking study, investigators demonstrated that B-vitamin supplementation (2.5 mg/d folate, 50 mg/d VB6 and 1 mg/d VB12) nearly completely prevented reduced mitochondrial DNA content and decreased DNA methylation through protecting against PM2.5-induced DNA hypomethylation. Meanwhile, these methyl group-supplying nutrients might minimize DNA hypermethylation by interacting with essential enzymes including *DNMTs* and *MTHFR* [72]. These findings point out that B vitamins might avert the loss of DNA methylation induced by air pollution, although this study was conducted as a short time (2 h) exposure with high PM2.5 concentration (250 μg/m^3^) in adults. As Lucock et al. mentioned, a study from Zhong et at. draws attention to the role of B-vitamin in exposomal factors, yet it is still premature to draw a conclusion [459]. Interestingly, Zhong et al. also reported such a vitamin B diet can mitigate the effects of PM2.5 exposure on cardiac autonomic dysfunction and inflammation [460].

### Diets vs. heavy mental

Dietary folic acid supplementation has been shown to prevent, at least in part, the adverse effects caused by environmental contaminant including chromium [461] and arsenic [462, 463]. Wang et al. conducted a study within workers from a chromate production plant and found that global DNA hypomethylation and DNA damages in blood were associated with decreased serum folate, suggesting folic acid supplementation may maintain genome stability and block cancer development in chromate sufferers [461]. Moreover, adequate folate has been shown to modify DNA methylation in peripheral blood leukocytes (PBL) [462] and *Alu* repetitive elements [463] of arsenic-exposed adults, suggesting a potential protective role of one-carbon metabolism nutrients in arsenic toxicity.

Except one-carbon metabolism nutrients and phytochemicals mentioned above, prenatal vitamin C [464–467], vitamin D [468–471], and certain polyphenols [59, 472–474] supplementation have been shown to maintain organismic normal growth and development, reduce susceptibility to disease, and prolong tumor latency through epigenetic regulation. All these epigenetic agents could be potentially used to counteract environmental toxicant-induced epigenome abnormity. It should be recognized that the investigations of prenatal nutrition intervention targeting environmental insults are still in the exploratory stage and more studies are needed.

## Potential considerations of prenatal nutritional intervention against environmental contaminants

### Windows of intervention

Early life, including germ cell differentiation and preimplantation of the embryo in the first trimester of humans, and infancy, is susceptible for external environmental stimulation to disrupt epigenome reprogramming. If exposed early, more serious consequences may occur compared with late gestation or adulthood exposures. Similarly, there are optimal windows of nutritional intervention to resist environmental insults. In-depth understanding of the relationship between dynamic change of the epigenome, environmental disturbance, epigenetics diet properties and disease susceptibly may lead to considerable progress in the epigenetic chemoprevention and pharmacological therapies [35].

### Global influence of epigenetics agents

As abovementioned, epigenetics diets usually exhibit global epigenetic modification such as DNMT inhibition and HDAC inhibition. Although numerous findings indicate that early-life nutrition supplementation reduces adverse effects of exposure to epigenetically toxic agents, some concerns are raised because of their potential, unpredictable targets in multiple genes by large-scale epigenetic perturbation, which are still unclear. There is promise that more targeted strategies will be developed and epigenetic therapies would be a powerful choice in clinical practice in the future [15].

### Multiple contaminants exposure

It is noteworthy that humans are often exposed to numerous environmental factors instead of a single contaminant. As detailed previously, most of the investigations only examined epigenome dysregulation caused by a single source of pollution. In developing nutritional strategies, therefore, the assessment of multiple contaminants, such as category, dosage, and duration, should be taken into consideration [114].

### Nutritional balance and combination

Nutritional balance is a noteworthy factor for early-life nutritional intervention. Otherwise, it is likely to have the opposite effect. As evidence of this, low maternal VB12 and high folate levels have been shown to increase obesity incidence and insulin resistance in offspring [458]. In addition, DeVita and Vincent reported that the combinatorial strategies have better therapeutic effect on cancers than treatment individually [475]. The most explored epigenetics drug combinatorial strategies are DNMT inhibitors and HDAC inhibitors [476, 477]. In line with this, we have been making progress by studying the interactions between dietary epigenetic-modifying compounds and combinatorial strategies in cancer research [268, 269, 276, 283, 409, 411, 478]. Given similar epigenome dysregulation caused by environmental toxicant exposure, combination addition of epigenetics diets could be a more promising approach to resist environmental disruption.

## Conclusion

Increasing evidence has indicated that prenatal dietary intervention may partially counteract adverse outcomes caused by exposures to environmental contaminants through averting epigenome dysregulation. Diseases, exposures, and specific genes-targeted approaches are urgently required for nutritional or pharmacologic interventions, since the epigenetic processes implicated in fetal adaptation to negative environmental stimulation still lack a comprehensive understanding. Moreover, time-, sex-, and genetic background-specific; dose-dependent;and global response to parental nutrition intervention, as well as a balanced nutrition regime against multiple pollutants, should be further investigated.

## Abbreviations

1,25-D3: 1,25-Dihydroxyvitamin D3
5caC: 5-Carboxylcytosine
5mC: 5-Methylcytosine
ACSL3: Acyl-CoA synthetase long-chain family member 3
ADP: Adenosine diphosphate
AHRR: Aryl hydrocarbon receptor repressor
Alu: *Arthrobacter luteus*
APC: Adenomatous polyposis
BDNF: Brain-derived neurotrophic factor
BPA: Bisphenol A
CGIs: CpG islands
CNTNAP2: Cortactin-associated protein-like 2
COMT: Catechol-*O*-methyltransferase
CYP1A1: Cytochrome P450 1A1
DDE: Dichloro-diphenyl-dichloroethylene
DDT: Dichloro-diphenyl-trichloroethane
DKK1: Dickkopf-related protein 1
DLGAP2: Discs large homolog-associated protein 2
DMR: Differentially methylated region
DNMT: DNA methyltransferase
DOHaD: Developmental origins of health and disease
EDCs: Endocrine-disrupting chemicals
EGCG: (−)-epigallocatechin-3-gallate
ESCs: Embryonic stem cells
FEBAD: Fetal basis of adult disease
GE: Genistein
GFI1: Growth factor independent 1
HAT: Histone acetyltransferase
HDAC: Histone deacetylase
hTERT: Human telomerase reverse transcriptase
ICM: Inner cell mass
IGF2: Insulin-like growth factor 2
ITCs: Isothiocyanates
JHDMs: JmjC-domain-containing histone demethylases
JmjC: Jumonji C
LINE-1: Long interspersed nuclear element
MBPs: Methyl-CpG-binding proteins
MetS: Metabolic syndrome
mtDNA: Mitochondrial DNA
MTHFR: Methylation of methylene tetrahydrofolate reductase
MYO1G: Myosin 1 G
NR3C1: Nuclear receptor subfamily 3 group C member 1
PAHs: Polycyclic aromatic hydrocarbons
PBDEs: Polybrominated diphenyl ethers
PBL: Peripheral blood leukocyte
PCBs: Polychlorinated biphenyls
PcG: Polycomb group
PDAC: Pancreatic ductal adenocarcinoma
PDLIM2: PDZ-LIM domain-containing protein 2
PFOA: Perfluorooctanoic acid
PGCs: Primordial germ cells
PM: Particulate matter
POPs: Persistent organic pollutants
PRDM8: PR domain containing 8
RSV: Resveratrol
RUNX3: Runt-related transcription factor
SAM: *S*-adenosylmethionine
Sat2: Satellite 2
Se: Selenium
SFN: Sulforaphane
SIRT1: Sirtuin 1
SULT2A1: Sulfotransferase 2A1
T2D: Type 2 diabetes
TCDD: 2,3,7,8-Tetrachlorodibenzo-p-dioxin
TET: Ten-eleven translocation
TNF-α: Tumor necrosis factor alpha
VB12: Vitamin B12
VDR: Vitamin D receptor
WA: Withaferin A
WHO: World Health Organization
